# PCR diagnosis of tick-borne pathogens in Maharashtra state, India indicates fitness cost associated with carrier infections is greater for crossbreed than native cattle breeds

**DOI:** 10.1371/journal.pone.0174595

**Published:** 2017-03-30

**Authors:** Sunil W. Kolte, Stephen D. Larcombe, Suresh G. Jadhao, Swapnil P. Magar, Ganesh Warthi, Nitin V. Kurkure, Elizabeth J. Glass, Brian R. Shiels

**Affiliations:** 1 Nagpur Veterinary College, Maharashtra Animal and Fishery Sciences, Nagpur, Maharashtra, India; 2 Institute of Biodiversity, Animal Health and Comparative Medicine, University of Glasgow, Glasgow, United Kingdom; 3 The Roslin Institute and Royal (Dick) School of Veterinary Studies, University of Edinburgh, Easter Bush, Midlothian, United Kingdom; Washington State University, UNITED STATES

## Abstract

Tick-borne pathogens (TBP) are responsible for significant economic losses to cattle production, globally. This is particularly true in countries like India where TBP constrain rearing of high yielding *Bos taurus*, as they show susceptibility to acute tick borne disease (TBD), most notably tropical theileriosis caused by *Theileria annulata*. This has led to a programme of cross breeding *Bos taurus* (Holstein-Friesian or Jersey) with native *Bos indicus* (numerous) breeds to generate cattle that are more resistant to disease. However, the cost to fitness of subclinical carrier infection in crossbreeds relative to native breeds is unknown, but could represent a significant hidden economic cost. In this study, a total of 1052 bovine blood samples, together with associated data on host type, sex and body score, were collected from apparently healthy animals in four different agro-climatic zones of Maharashtra state. Samples were screened by PCR for detection of five major TBPs: *T*. *annulata*, *T*. *orientalis*, *B*. *bigemina*, *B*. *bovis and Anaplasma* spp.. The results demonstrated that single and co-infection with TBP are common, and although differences in pathogen spp. prevalence across the climatic zones were detected, simplistic regression models predicted that host type, sex and location are all likely to impact on prevalence of TBP. In order to remove issues with autocorrelation between variables, a subset of the dataset was modelled to assess any impact of TBP infection on body score of crossbreed versus native breed cattle (breed type). The model showed significant association between infection with TBP (particularly apicomplexan parasites) and poorer body condition for crossbreed animals. These findings indicate potential cost of TBP carrier infection on crossbreed productivity. Thus, there is a case for development of strategies for targeted breeding to combine productivity traits with disease resistance, or to prevent transmission of TBP in India for economic benefit.

## Introduction

Tick-borne pathogens (TBP) are considered to be one of the major hindrances to productivity and health of livestock, globally [1–3]. The four main pathogens responsible for these losses are the tick borne protozoa, *Babesia* and *Theileria*, and the tick-borne rickettsial disease pathogens, *Anaplasma* and *Ehrlichia*. In developing countries like India, TBP spp. can impose considerable economic loss on large and smallholding livestock productivity farming systems: the resulting diseases causing high mortality rates, reduced milk production and loss of body condition [1, 4].

The impact of bovine theileriosis on cattle productivity in India is considerable. The *Theileria* species that infect bovines in India are *T*. *annulata* and *T*. *orientalis*, and both species are transmitted by *Hyalomma anatolicum*. While *T*. *annulata* is widely considered to be more pathogenic and associated with greater economic loss, outbreaks of disease due to the more benign *T*. *orientalis* have been reported recently [5, 6] and may represent a hidden burden to livestock productivity in regions where this species is endemic. A total 190 million cattle and 108 million buffaloes are currently reared in India [7], with (approximately) 39 million crossbred cattle being most at risk from tropical theileriosis caused by *T*. *annulata*. Annual losses of the livestock industry due to tropical theileriosis alone were estimated to be US$ 384.3 million in 2003, and further economic loss is incurred due to babesiosis, tick worry and the embargo on importation of exotic breeds [8].

Babesiosis or tick fever is a disease caused by intraerythrocytic protozoan parasites within the genus *Babesia*. The main pathogenic species is thought to be *B*. *bigemina* but *B*. *bovis* infection has also been reported in India [9–12] and Maharashtra state [13]. These species are primarily transmitted by *Rhipicephalus (Boophilus) microplus*, and bovine species under stress from other diseases are potentially susceptible to infection [14]. *Babesia* infections in India have been reported across indigenous, crossbred cattle and buffaloes with an associated annual economic loss of more than US$ 57.2 million [4, 15].

Anaplasmosis in India is primarily transmitted by *Rhipicephalus (Boophilus) microplus*. The pathogenic *Anaplasma marginale* has been reported in multiple states of India, including Odisha, Uttar Pradesh, Punjab, Haryana, Tamil Nadu and Karnataka [4]. Ehrlichiosisis is caused by *Ehrlichia ruminantium* in ruminants, and is transmitted by ticks within the genus *Amblyomma*. Both anaplasmosis and ehrlichiosis have been reported to cause significant economic losses in the livestock industry of Caribbean countries and other sub-tropical regions of the world [16]. However, there is no significant scientific literature on the spread of bovine ehrlichiosis in India, with only one report published [17].

Losses due to overt clinical disease can be diagnosed following identification of tick borne pathogens, usually by microscopic examination of blood smears. However, pathogen species can be misidentified and due to this, and a lack of sensitivity, the level of carrier animals can be underestimated by microscopy. This lack of information is relevant for a number of reasons: firstly, accurate speciation aids in implementation of the best available control strategy; secondly, knowledge of the reservoir of infection is required to estimate the potential risk of repeated transmission in any one geographical area, and the risk associated with livestock movement from one area to another; thirdly, recent work has shown that, for tropical theileriosis at least, the economic impact of the carrier state can be as significant as clinical disease [18] and so estimation of the level of economic loss requires assessment of the number of sub-clinical, carrier infections in addition to animals suffering from overt disease.

With the introduction of PCR based molecular techniques, the sensitivity, specificity and reproducibility for detection of pathogens associated with TBD has improved. Thus, both single PCR and semi-nested PCR assays have been validated as more sensitive and specific for detecting TBD infections [4]. In this study we aimed to utilize PCR methodology to obtain a preliminary assessment of the potential burden of TBP carriers in the state of Maharashtra in India, as its bovine population has never been screened for these pathogens by a molecular based assay. To investigate whether the threat of infection was variable across regions, the PCR-based survey was conducted in four of the nine different agro-climatic zones of Maharashtra (Table 1). Prevalence data can be used to assess whether a region is a source for the spread of TBD, or is at risk from outbreaks of clinical disease. Moreover, together with information on differences between hosts, prevalence data can be used to examine associations between intrinsic and extrinsic factors that determine consequences of disease. For example, disease severity and economic loss may be influenced in large part by the genotype of the host, as well as the regional burden of infection. Association analyses of breed type and physiological costs of infection may be particularly useful in the Indian context where indigenous *Bos indicus* (native) cattle breeds are farmed together with the practice of rearing taurine/indicus crossbreeds to improve productivity in the face of pathogen challenge. There is scant information about relative disease resistance in these bovine species and although it appears that native buffalo and cattle breeds are more resistant to disease than “exotic” high milk producing cattle, it is unclear to what extent crossbred cattle retain such disease resistance/tolerance traits [19]. By sampling a cross section of buffalo, native and crossbred cattle in both uninfected and carrier-infected TBP states and combining this data with production-linked traits we aimed to test for specific differences in the outcome of infection. Such information is important for examining the possible cost of the carrier state in both large and smallholder farming practises and assessing whether genetic improvement or prevention of TBP transmission would be of economic benefit to livestock producers in India.

**Table 1:** List of number of samples collected from various district and zone.

Sr. No.	Name of the Zone	District	Number of samples		Total
			Cattle	Buffalo	
1.	Eastern Vidarbha zone (EVZ)	Gondia	122	31	153
2.	Scarcity zone (SCA)	Ahmednagar	48	-	48
		Nashik	354	-	354
3.	Moderate rainfall zone (MRZ)	Nagpur	108	50	158
4.	Assured rainfall zone (ARZ)	Akola	125	47	172
		Amravati	39	-	39
		Latur	67	61	128
**Total**			**863**	**189**	**1052**

## Materials and methods

### Blood sample collection

Blood samples were collected from four different agro-climatic zones represented by 7 districts of Maharashtra state (Table 1). These zones were selected based on two important factors: the higher level of milk production by cattle and water buffalo reared in these zones and the rainfall gradient, with the four zones representing different climatic conditions. The reason for selecting regions of higher milk production is that TBP may be a particular concern for food production and security, and these zones represent important areas for cattle/buffalo productivity where maximal production is expected from apparently healthy animals. The zones are: Eastern Vidarbha Zone (ERZ), average rainfall of 950 to 1250 mm, with 1700 mm extreme rainfall on the eastern side of the zone (Gondia district) for 59 days; Moderate rainfall zone (MRZ), average rainfall of 1130 mm; Assured rainfall zone (ARZ), average rainfall of 700 to 900 mm covering 75% of the districts under this zone; and the Scarcity zone (SCA) that has a very low rainfall (i.e. 750 mm in monsoon) displaying a bimodal pattern of unpredictable occurrence, resulting in drought every third year. A map locating these zones and information on climate conditions (including average rainfall) can be found at: http://www.mahaagri.gov.in/CropWeather/AgroClimaticZone.html. Blood samples were randomly collected from February to August 2015 (Table 1) from 1052 apparently healthy bovine animals (no sign of pyrexia, anorexia, or reduced milk production) belonging to all age groups. Breakdown of the animals was as follows: 189 buffaloes (189 female, 0 male); 588 crossbred *Bos taurus X Bos indicus* (574 female, 14 males); and 275 native breed *Bos indicus* (221 female, 54 male). In these populations crossbred cattle always contain a 50:50 ratio of *Bos taurus*:*Bos indicus*. Two ml of blood obtained from the jugular vein were collected in EDTA vacutainer tubes. Samples were labelled, carried to the laboratory at 4°C and stored at -20°C prior to isolation of DNA. All animals had their body condition scored between 1–5 using visual guidelines provided by Queensland Government, Australia (see S1 Fig) together with manual palpation: **Body condition score 1:** Backbone prominent, Hips & shoulder bones prominent, Ribs clearly visible, Tail head area recessed, Skeletal body outline; **Body condition score 2:** Backbone visible, Hips & shoulder bones visible, Ribs visible faintly, Tail head area slightly recessed, Body outline bony; **Body condition score 3**: Hip bones visible faintly, Ribs generally not visible, Tail head area not recessed, Body outline almost smooth; **Body condition score 4:** Hip bones not visible, Ribs well covered, Tail head area slightly lumpy, Body outline rounded; **Body condition score 5**: Hip bones showing fat deposit, Ribs very well covered, Tail head area very lumpy, Body outline bulging due to fat. In Indian dairy cattle and buffalo, animals with better body condition scores are generally more productive milk producers [20]

### Sample preparation for PCR

Collected samples were treated with lysis buffer and Proteinase K to remove potential inhibitors of PCR present in the blood. For each sample, 500 μl of blood was aliquoted into a microfuge tube filled with 1ml lysis buffer (0.22% NaCl, 0.015% saponin, 1 mM EDTA, pH 7.5). Tube contents were mixed and centrifuged at 9,300Xg for 5 minutes and the resulting supernatant discarded: pellets were resuspended in 750 μl of lysis buffer, centrifuged and supernatants discarded; this process was repeated until the pellet was clear of haemoglobin. The final pellet was resuspended in 100 μl of 50 mM KCl, 10 mM tris-HCl pH 8.0, 0.5% Tween 20 and 100 μg proteinase K per ml. The tubes were then incubated in a water bath at 56°C for 2 hours after which they were immediately stored at -20°C. 1μl of the lysate was used as a template for PCR reactions.

### PCR assay for diagnosis of tick borne pathogens and validation by sequencing

A semi-nested PCR was used for the detection of TBP based on methodology and primer sets previously developed and validated with species specific DNA samples [21–23] (S1 Table). A primary reaction was carried out using two universal primer sets designed against the 18S rRNA gene for *T*. *annulata*, *T*. *orientalis*, *B*. *bigemina and B*. *bovis* or the 16S rRNA gene for *Anaplasma* spp. and *E*. *ruminantium*. Each PCR reaction was carried out in a 25 μl reaction volume containing 10 pmol/μl of each primer, 1 μl of template (DNA in sample lysate) and 12.5 μl of 2X GoTaq^®^ Green Master Mix (Promega Corporation). Negative controls consisted of water or *E*. *coli* DNA. Thermal cycler conditions for the primary universal reactions were: 35 cycles of 30 sec at 94°C, 30 sec at 57°C and 40 sec at 72°C. For species specific semi-nested PCR, primary reaction samples generating positive PCR products were diluted 1:100 and 2 μl of diluted primary reaction mix was used as a template for the semi-nested secondary reaction. Thermocycler conditions for the nested PCR reactions were similar to primary reaction except the annealing temperature for the *T*. *annulata* and *T*. *orientalis* primer sets were 48°C for 90 sec and 55°C for 90 sec, respectively. PCR products were separated by gel electrophoresis on a 1.5% agarose gel and visualized by ethidium bromide stain under UV using a Gel Doc (Syngene) apparatus. The primers used in the studies were tested for specificity previously using combinations of field isolates and standards but as an additional confirmation, positive PCR reactions were validated for amplification of the species-specific target gene by direct sequencing of two representative amplicons for each species-specific primer set. Amplified DNA samples were sent for direct sequencing (Eurofins Scientific India Pvt. Ltd.) and sequences were validated by NCBI BLAST searches (https://blast.ncbi.nlm.nih.gov/Blast.cgi) to validate identity to the target species as the top hit.

### Statistical analyses

Modelling approaches were used to determine whether any observed differences in parasite prevalence or host body condition were statistically significant. Dataset can be found in supporting infromation (S1 Dataset). For a primary investigation of the impact of host species (Buffalo versus all cattle types) on parasite prevalence, we first constructed a simple logistic regression model using information from all sampled animals. Firstly, binary data on the presence or absence of each of five tick-borne pathogens were analysed to determine whether there were statistically significant differences in disease prevalence among host species (buffalo versus cross breed *and* native cattle breeds), sex or location. Next, we examined in more detail how intrinsic differences between hosts at the individual level impacted the prevalence of different TBD. As host type (cattle versus buffalo) was a significant fixed effect in many of the preliminary models (see Results), and our data contained far more cattle than buffalo (864 versus 189), we decided to use a shortened cattle only dataset for these analyses. Thus, for all cattle (male and female) only, we constructed models for including breed type (native *B*. *indicus* versus cross-bred *B*. *taurus X B indicus*), sex, region and the interaction of sex*breed type to test for differences in the distribution of diseases among locations and breed type.

A mixed modelling approach was used to examine the contribution of other fixed effects while including Zone (location effect) as a random factor to control for non-independence of animals from the same geographic origin. All of the zones sampled contained large numbers of female cattle, containing both cross-bred and indigenous breeds, and this subgroup of 795 individual female cattle offered the best data set for a non-biased comparison between infection status, regions, breed and age. A Generalised Linear Mixed Model (GLMM) approach using the MCMCglmm package for R (version 3.1.0; R Development Core Team 2011) was adopted. Frequentist mixed models are not always effective for the analysis of multivariate or non-normally distributed variables [24], as is the case for binary and count data from the sampled cattle. Bayesian methods provide an alternate and flexible framework for the analysis of hierarchical data. We used the MCMCglmm package (version 2.21 [25]) to fit models within a Bayesian Monte Carlo Markov Chain (MCMC). MCMCglmm uses Bayesian numerical integration methods, producing reliable confidence intervals on variance components from small data sets, allowing the modelling of count data with over-dispersed distributions [26]. To reduce the complexity in the data and avoid the problems of multiple comparisons we created two new variables: Apicomplexan status (infected or not with any of the four apicomplexan species) and *Anaplasma* spp. status (infected or not with either of the two bacterial species). We also created three age classes based on biological and veterinary reasoning. Age class 1: 0–2 years old, reflecting immature, non-breeding, non-milk producing animals; Age class 2: 3–9 years, reflecting animals of a milk producing and breeding age; Age class 3: 9–14 years olds, reflecting old animals unlikely to serve a productive role and co-grazing with younger cattle. We modelled TBP as a dependent variable with a categorical distribution. Total number of infections was also counted and modelled with a poisson distribution. Zone was entered as a random factor in every model to account for non-independence of data from similar agro-climatic locations. To account for differences in susceptibility to disease with age in cross breeds versus indigenous cattle breeds we entered: breed, age-class, farm vaccination status -vaccinated or not (vaccination in this region of India is typically for Haemorrhagic Septicaemia, Black Quarter, and Foot and Mouth Diseases), acaricide status—treated or not; breed type- indigenous versus cross breed; and the interaction of breed type*age-class. To improve the performance of these models, we used an expanded prior with the variance of the fixed effects set to 1, since we were dealing with binary distributed parameters.

To test for potential parasite specific, age specific, or breed type specific differences in the outcome of infection in carrier animals, we used MCMCglmm to model body condition scores for all female cattle. Body condition was modelled with a normal distribution. As for the MCMCglmm models for disease prevalence, we chose to model apicomplexan status and *Anaplasma* spp. status as fixed factors, rather than individual parasites. Next we entered infection status, breed type, age and all possible two-and three-way interactions to the model. The model was simplified by removing non-significant interaction terms until only significant terms remained, to partition the contribution of infection with different parasite genera, host breed types and ages on body condition. This model also contained Zone as a random factor, and used an expanded prior, though the variance of the fixed effects was not constrained.

### Ethical statement

This study was carried out in strict accordance with the recommendations of the Veterinary Council of India, and all work was overseen by their staff. Ethical approval was not required by the ethical committee for performing animal experiments (Institutional Animal Ethics Committee of Nagpur Veterinary College), as: 1. the survey was conducted by government official veterinary physicians in village farms within their jurisdiction, 2. samples were taken as part of the standard course of veterinary inspection to determine presence of infections detrimental to animal health in India and 3. in India, ethical approval is not required for survey work conducted for the benefit of livestock welfare or improved farming practice, as in this case. No animals were housed or harmed as part of the survey, and every care was taken to minimise any suffering of the animals during the brief handling period.

## Results

### Pathogen prevalence across different agro-climatic zones

Primers used for the semi-nested PCR assay were previously demonstrated to be specific for the apicomlexan parasites tested but not for *A*. *marginale*[21]. These findings were supported in the current study, as one major product of the predicted size per primer set was observed across field samples(S2 Fig). In addition species validation was performed by sequence analysis of two representative amplicons for each primer set. Blast analysis showed >98% identity with NCBI reference sequence (and top BLAST hit) for two amplicons designated as *T*. *annulata*, *T*. *orientalis*, *B*. *bovis*, *B*. *bigemina* and >98% for *at least two different Anaplasma* spp (S2 Table); the amplicon obtained with *E*. *ruminantium* primers, however showed hits with *A*. *marginale* (>93%): indicating an absence of *E*. *ruminantium* in the regions surveyed and cross annealing of the *Ehrlichia* primers to *Anaplasma* DNA. Hereafter, the results from the *Anaplasma* and *Ehrlichia* primers are combined and denoted as *Anaplasma* spp. Under the conditions used in this study (PCR detection limited to 500 μl blood sample), the PCR assay was deployed to test for the presence of *T*. *annulata*, *T*. *orientalis*, *B*. *bovis*, *B*. *bigemina* and *Anaplasma* spp. in cattle from four agro-climatic zones of Maharashtra. Table 2 shows the raw prevalence data (%) for the 1052 bovine animals sampled in the four agro-climatic zones. Preliminary observation of the data is suggestive of geographical differences in prevalence of TBP: Of the four agro-climatic zones, the highest percentage of TBP infected animals was observed in the SCA (65.2%) followed by the ARZ, EVZ and the MRZ (Table 2), respectively. The statistical significance of these apparent differences is tested below. General findings were that *Anaplasma* spp. was the most common pathogen detected (53.3% of all bovine animals infected), while *B*. *bigemina* was the least common (1.3%). Co-infections were common (S3 Table): for example, *T annulata* was found more commonly in double (90 animals) or triple (47 animals) infections than in single infections (43 animals), with co-infection involving *T*. *orientalis* representing 70 of 166 (42%) total *T*. *annulata* infections. Of the 561 samples positive for *Anaplasma* spp., most were single infections, but around 10% were double or triple infections with Apicomplexa spp. We found 17 animals carrying 4 or more TBP, and all were Holstein-Friesian crossbred cattle from SCA.

**Table 2:** PCR screening results for different parasites in four different agro-climatic zones of Maharashtra state, India. Nb. Column totals for each individual TBP include all types of infections (e.g. 7 positive T annulata may have occurred singly, or in co-infections with other parasites).

Agro-climatic zone	Total samples	Number of positive samples for					
		≥1 parasite	*T*. *annulata*	*T*. *orientalis*	*B*. *bigemina*	*B*. *bovis*	*Anaplasma* spp.
Eastern Vidarbha Zone	153	96 (62.7%)	7 (13.2%)	3 (1.9%)	0 (0.0%)	0 (0.0%)	92 (60.1%)
Moderate Rainfall Zone	158	67 (42.4%)	33 (20.9%)	17 (10.8%)	4 (2.5%)	0 (0.0%)	33 (20.8%)
Assured Rainfall Zone	339	213 (62.8%)	100 (29.5%)	40 (11.8%)	3 (0.9%)	5 (1.5%)	182 (53.1%)
Scarcity Zone	402	262 (65.1%)	26 (6.5%)	32 (7.7%)	7 (1.7%)	27 (6.7%)	254 (63.2%)
**Total**	**1052**	**638 (60.6%)**	**166 (15.8%)**	**92 (8.7%)**	**14 (1.3%)**	**32 (3.0%)**	**561 (53.3%)**

### Modelling host type and regional differences in parasite prevalence

To test using statistical approaches whether, as suggested by the primary data (Table 2), that differences in parasite prevalence is significantly mediated by intrinsic host factors (e.g. species/ sex) or extrinsic factors like agro-climatic zone, simple logistic regression models were generated. These preliminary models generated strong evidence for differences mediated by host species (buffalo versus cattle) in the prevalence of some tick borne pathogens (TBP), but not others. There was no significant difference between cattle or buffalo in *T*. *annulata* or either *Babesia* spp. (*T*. *annulata* Wald’s W = 0.47, p = 0.49; *B*. *bovis* W = 0.12, p = 0.72; *B*. *bigemina* W = 0.13, p = 0.75), but for*Anaplasma* spp. and *T*. *orientalis* (*T*. *orientalis* W = 11.1, p = 0.001; *Anaplasma* W = 10.1, p = 0.01) cattle were significantly more likely to carry infection than buffalo. If co-grazing buffalo were a major reservoir for TBP infection in cattle, as is the case for *Theileria parva* [27] a higher prevalence in buffalo might be predicted. Our results do not indicate that this is the case for any of the pathogens tested for.

To attempt to account for discrepancy between sample sizes of buffalo and cattle we analysed data only from all (male and female) cattle, the majority of our dataset. In cattle, all of the parasites showed geographical (zone) variability in their prevalence (a significant effect of location), except *B*. *bigemina* (see Table 3), for which the sample size of detected positives was probably too small to be conclusive. This highlights that some aspect of geographical region contributes to the epidemiology of TBP in Maharashta state at a broad scale. Furthermore, the simple logistic regression indicated that prevalence of *T*. *orientalis* is dependent on host breed type, with native breeds having an apparently higher prevalence than crossbreeds.

**Table 3:** Output from logistic regression of factors impacting prevalence of different parasites in cows in Maharashtra.

	*T*. *annulata*		*T*. *orientalis*		*B*. *bovis*		*B*. *bigemina*		*Anaplasma* spp.	
	Wald Chi Sq	P-value	Wald Chi Sq	P-value	Wald Chi Sq	P-value	Wald Chi Sq	P-value	Wald Chi Sq	P-value
Intercept	85.76	0.001*	38.67	0.001*	13.00	0.001*	0.000	0.99	0.80	0.370
Breed Type	0.57	0.451	7.29	0.007*	0.003	0.956	0.203	0.65	2.54	0.111
Zone (location)	50.62	0.001*	9.22	0.026*	3.85	0.05*	0.821	0.84	60.43	0.001*
Sex	0.82	0.363	4.64	0.031*	0.000	0.99	1.354	0.25	0.017	0.898

Statistically significant fixed effects are marked *

### Intrinsic and extrinsic host factors impact prevalence of TBP

Given the differences in productivity and susceptibility associated with breed, we were particularly interested in investigating breed type, and breed type*age interaction as potential mediators of parasite prevalence. Our simplistic regression models revealed that breed type, sex and location can all impact on the prevalence of TBP in cattle, with apparently significant differences mediated by host breed type in the prevalence of *T*. *orientalis*, and a similar non-significant trend for *Anaplasma* spp. However, the distribution of crossbreeds and native breeds across zones was unequal: ARZ had by far the largest sample size of native breeds (185 of 221) and the highest prevalence of *T orientalis* in cattle, so these results should be taken cautiously.

**Fig 1:**
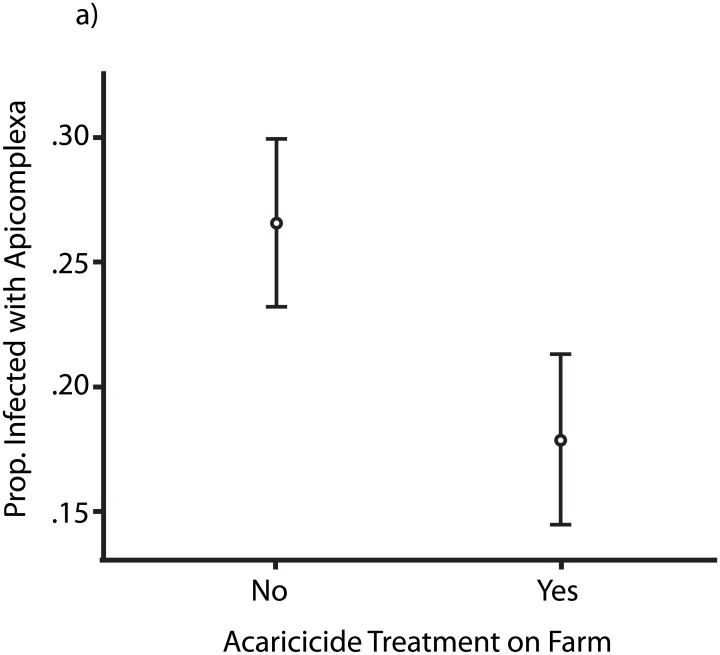
Proportion of female cattle (+/- standard error) infected with any apicomplexan parasite according to acaricide treatment. The data for this analysis comes from a subset of farms where both breed types were kept.

Preliminary attempts at more advanced mixed modelling showed that the dataset contained strong autocorrelation between variables (such as the example for breed type and location for *T*. *orientalis* given above) and poor mixing of the MCMCglmm. We were most interested in impacts of breed, but in some cases farms or zones only contained one breed of cattle, or one breed was far more common than the other. To investigate breed type differences (cross versus native) specifically we reduced the dataset to locations where both types of (female) cattle were kept concurrently (294 individuals, 3 farms, 3 Zones). Results are shown in Table 4. No evidence of any impact of breed type or age on the prevalence of apicomplexan parasites or *Anaplasma* spp. was found. However, the prevalence of apicomplexan parasites was significantly lower in (female) cattle from farms where acaricide treatments were used (Table 4 and Fig 1). Conversely, acaricide treatment did not appear to impact *Anaplasma* spp. prevalence, implicating a difference in the transmission of these genera of pathogens, perhaps mediated by differences in number of competent vector species/infectivity.

**Table 4:** Output from MCMCglmm explaining different aspects of infections status, according to host age and breed type, and farm practice. Data came from subset of female cattle where native and crossbreeds cohabited the same farms and the table reports values from the final model following removal of non-contributing fixed effects and interactions.

	Posterior Mean	L-95% C.I.	U-95% C.I	Effective Sample Size	pMCMC
**Total No. Infections**					
place	14.5	1.1x10-5	9.25	521	
(Intercept)	0.29707	-2.58405	4.04922	1156	0.872
Breed type	-0.11105	-0.56548	0.32472	2000	0.618
age class	-0.21617	-0.58534	0.17283	2000	0.247
acaricide treatment	-0.43909	-1.36708	0.49036	1603	0.364
vaccination	-0.03278	-0.90194	0.78611	1685	0.962
**Apicomplexan Infection Status**					
location	43.06	1.8x10-7	157	315	
(Intercept)	-0.76257	-6.91175	5.12882	2719	0.547
Breed type	-0.09615	-0.77691	0.68626	1680	0.799
age class	0.22371	-0.42928	0.88734	1682	0.486
acaricide treatment	-1.66745	-3.32166	-0.01827	806	0.026*
vaccination	0.56982	-0.90163	1.96086	1856	0.443
**Multiple Infection Status**					
location	28.04	2.45x10-6	94.06	463	
(Intercept)	3.594	-2.6346	10.2064	2870.9	0.215
Breed type	-3.1726	-6.0226	-0.5286	1551.1	0.02*
age class	-2.6049	-4.5877	-0.5945	1809.6	0.011*
acaricide treatment	-0.5001	-2.0654	1.0522	971.5	0.567
vaccination	1.5511	-0.5071	4.1432	889.5	0.121
Breed type*age class	1.37	0.1214	2.7783	1717.2	0.044*
***Anaplasma* spp. Infection Status**					
Location	127.9	0.45	502.4	134	
(Intercept	2.4181	-13.1227	12.2393	1481	0.433
Breed type	-0.1538	-0.9581	0.5821	1730	0.713
age class	-0.4809	-1.1411	0.1403	1839	0.135
acaricide treatment	-0.2311	-1.5223	1.1117	1676	0.741
vaccination	-2.367	-4.4194	-0.6101	1193	0.003*

Statistically significant fixed effects are marked *.

**Fig 2:**
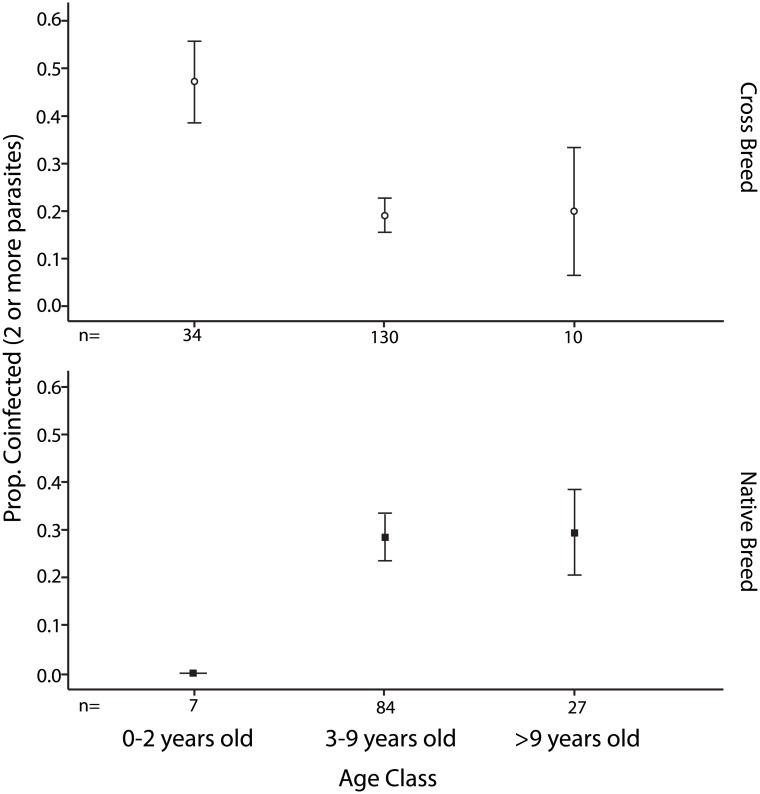
Proportion of TBP co-infections (+/- standard error) in female cattle by age class and breed type (crossbred upper panel, native breed lower panel). The data for this analysis comes from a subset of farms where both cattle breed types were kept.

A significant host age*breed type effect on the occurrence of multiple infections (animals infected by at least two parasites of any genera) was also indicated. Plotting the data shows that this effect is driven by differences in immature animals (< 2 years old). In this case, younger crossbreeds appear more susceptible to coinfections than younger native breeds (Fig 2). However, it is important to note there was a discrepancy in sample sizes for young animals.

### Impact of cattle breed type and infection status on body condition

**Fig 3:**
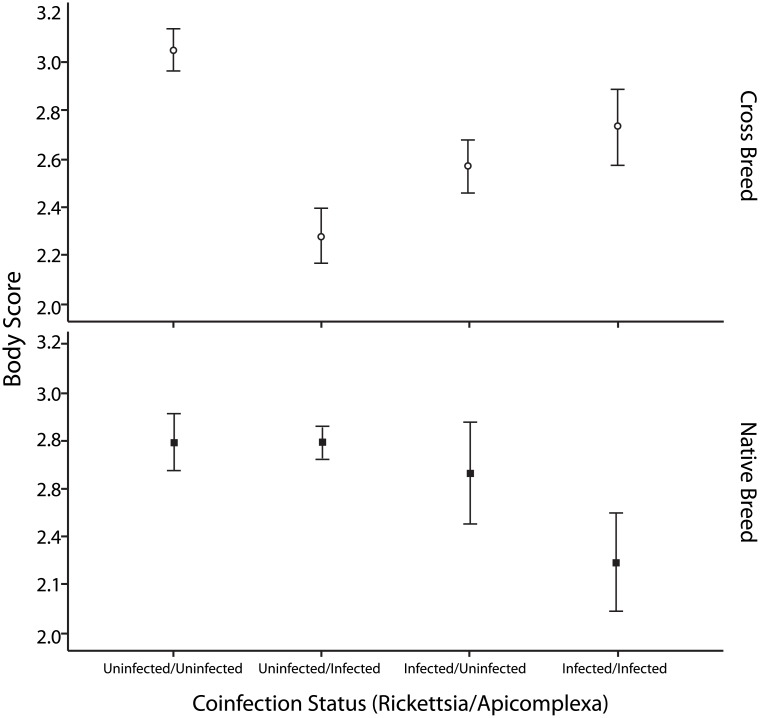
Mean (+/- standard error) body condition of female cattle of different breed types (cross breeds upper panel, native breeds lower panel) in relation to apicomplexan and *Anaplasma* spp. infection status. The graph shows crossbreeds in the upper panel and native breeds in the lower panel.

The infected animals monitored in the study were in a carrier state, which has been demonstrated previously to impact productivity at a level comparable to that of overt clinical disease for crossbred animals in Tunisia [18]. Therefore, we investigated whether we could uncover any breed type specific impact of infection on body condition scores. Using only the subset of data outlined above to best compare breed type effects we found a significant three-way interaction of apicomplexan infection, *Anaplasma* spp. infection and breed on body condition (Table 5). This data is cross sectional rather than longitudinal, but if we consider animals free of all infections (uninfected-uninfected) as a surrogate baseline, then the data (see Fig 3) indicates that for native breeds of (female) cattle, only mixed infections of apicomplexa together with *Anaplasma* spp. lead to a significant reduction in body condition (difference in mean body score = 0.5 for coinfection versus uninfected state). In contrast, for crossbred (female) cattle apicomplexan only infections (difference in mean body score = 0.72 versus uninfected state), *Anaplasma* spp only infections (difference in mean body score = 0.48 versus uninfected state) and double infections (difference in mean body score = 0.32 versus uninfected state) are all associated with reduced body condition versus uninfected/uninfected state, with the largest difference mediated by apicomplexan only infections. The significance of reduced body score at this scale to productivity and economic loss requires further study. Nevertheless, this data lends support to the premise that crossbreeds are potentially more susceptible to the consequences of TBP carrier infection than native breeds.

**Table 5:** Output from MCMCglmm testing the impact of infection status, age and breed type on body condition in female cattle. Data came from subset of female cattle where native and crossbreeds cohabited the same farms.

	Posterior Mean	l-95% CI	u-95% CI	eff.samp	pMCMC
location	8.73	0.002	37.97	318	
(Intercept)	3.02203	-0.096168	5.412611	2273	0.045*
Breed type	-0.094808	-0.563169	0.387545	2000	0.688
Age	-0.033788	-0.132397	0.064107	2000	0.494
Apicomplexa	-0.669919	-1.662902	0.226976	1858	0.168
*Anaplasma* spp.	-0.942394	-1.500202	-0.367728	2000	0.002 **
Breed type*Apicomplexa	0.123664	-0.539142	0.736539	2026	0.697
Breed type*Anaplasma spp.	0.321334	-0.050538	0.751228	2000	0.115
Breed type*Age	0.026466	-0.042187	0.089267	2000	0.439
*Anaplasma* spp.*Apicomplexa	1.681228	0.549235	3.166116	1785	0.012 *
Age*Apicomplexa	-0.005787	-0.087528	0.072539	2000	0.867
Age*Anaplasma spp.	0.045313	-0.020481	0.111914	2000	0.177
Breed type*Apicomplexa *Anaplasma spp.	-0.925169	-1.820479	-0.012415	1787	0.046 *

Statistically significant fixed effects are marked *.

## Discussion

The burden of tick borne disease to livestock productivity in countries such as India is a significant one. The impact is felt by large-scale production systems but is potentially even more devastating for smallholding rural farmers and communities. In addition to clinical episodes of TBD, unknown losses to productivity will also occur in animals that appear healthy but are infected. Moreover, these animals play a critical role in the epidemiology of TBD. The local environment is also a critical epidemiological factor, because while various tick species have been reported to transmit TBP to susceptible bovine animals in India [4], the relative abundance of those that act as vectors will differ depending on climatic requirements. To obtain information on the continuing risk of TBD to livestock, information on the prevalence of infection and knowledge of both intrinsic and extrinsic factors that influence this prevalence is required. To begin to assess these factors we performed a PCR based survey of the prevalence of major TBP in healthy cattle in four agro-climatic zones in Maharashtra state, India. Modelling was performed on the survey data set to investigate factors that can influence this prevalence and the outcome of infection.

Results from the PCR assay demonstrated that apart from *E*. *ruminantium* all TBP tested for are present in Maharashtra. The level of the infection with *Anaplasma* spp, in general, was found to be significantly greater than the other TBP across all four zones (see Table 2) versus. There are a number of postulations that can be put forward to explain this higher prevalence, the simplest being a greater number of competent tick vectors. *Anaplasma* spp. can be transmitted by at least 20 ticks species, mechanical transmission via by biting flies has been reported [28] and iatrogenic transmission is also possible [29]. *Anaplasma* is also associated with a long term/life long carrier state [30], and so the probability of detecting positive animals, particularly in areas of endemic instability, is increased. Despite the high prevalence, the level of infection varies across zones, with the difference in level of detectable infection being greatest between MRZ and SCA (28% and 62%, respectively). Clinical episodes of disease could occur, therefore, if adult naïve animals were moved from a region of low prevalence to one of high and then challenged with pathogenic *A*. *marginale*.

*Babesia* parasites were detected at low levels of prevalence (4.3% overall). The most notable finding was the detection of *B*. *bovis* in the SCA zone (6.7%), relative to no positive samples in EVZ or the MRZ and a lower number of positives in the ARZ. This is the first time *B*. *bovis* infection has been identified in Maharashtra state using PCR. It should be noted that the carrier state for *B*. *bovis* can be represented by very low parasitaemia. Therefore, based on the methodology adopted (PCR amplification from 500 μl) the number of carrier infections for *B*. *bovis*, in particular, (but all TBP at < 1 per ml) are potentially underrepresented. Thus our results, as in previous studies, are based on the sampling volume utilized and not the absolute limit of detection. The level of *B*. *bovis* prevalence indicates that a situation of enzootic instability is most likely, with low levels of challenge promoting the occurrence of naïve adult animals that are at risk from disease if challenge subsequently increases. The reason for an apparent higher level of infection in a dry zone (SCA) compared to a wetter zone (EVZ) is not clear, and it should be noted that the highest number of samples were taken in the scarcity zone, skewing the probability of detecting infections present at low prevalence. However, a similar situation was reported for Sri Lanka [31]. One possibility is that a coordinated challenge by infected ticks occurs after a period of rain in the scarcity zone, together with a carrier state that presents low levels of infection or is transient. The situation for *B*. *bigemina* was similar to *B*. *bovis*, except it was detected in 3 zones and did not show as marked an increase in the scarcity zone.

The prevalence data for *Theileria* species appear to present a contrasting pattern to the other TBP, since the % of infected animals was significantly greater in the ARZ and MRZ, relative to the EVZ and SCA. A simplistic explanation is that this may indicate that zones where *Theileria* is more prevalent are more suitable for the *Hyalomma* tick vector, although this is not supported by a preliminary survey on relative abundance of tick species across the 4 zones where the relative abundance of *Hyalomma* was similar, except in the SCA (see S4 Table). In the absence of regional confounding factors, it could be predicted that zones associated with higher rainfall are a greater risk (nearly one third of animals infected in the ARZ) for tropical theileriosis, with crossbreed and young animals the most likely to suffer from clinical disease [32].

Susceptibility to, or outcome of, TBP infection between exotic, crossbreed and indigenous breeds of cattle is extremely important in the Indian context given the low resistance of highly productive *Bos taurus* cattle to TBD [33]. However, the extent to which breed, or any other intrinsic host effect, contributes to the probability of becoming a TBP carrier animal and/or impacts on productivity are less well understood. Using the data collected we examined whether we could detect differences in the incidence of carrier infection with TBP, or the consequences of infection, between Indian native and crossbreed cattle. No evidence for an association of host breed type with prevalence of any individual parasite species was found. However, a breed type effect on the incidence of coinfection was indicated by the fact that young crossbreed cattle were more likely than native breeds, or older crossbreeds, to harbor infections with at least two different TBP species. In addition, crossbreed animals appeared to be more likely to harbor multiple co-infections. Overall though, these results indicate that the chance of becoming infected by a given TBP is probably not driven to a large degree by cattle breed type in Maharashtra.

Breed type, however, does impact on the consequences of being a carrier for TBP. As shown in Fig 3, body condition score is only reduced in native breeds when animals were co-infected by *Anaplasma* spp. and apicomplexan parasites. This suggests that in native breeds, carrier infection with either apicomplexan or *Anaplasma* pathogens alone might be insufficient to impact body condition. In contrast, the body condition of crossbreed animals differed between uninfected and those infected with either apicomplexan or *Anaplasma* pathogens alone. Indeed, the biggest difference between groups was between uninfected and apicomplexan parasite infected animals for crossbreeds and the biggest difference in body score was between apicomplexan carrier crossbreeds and uninfected crossbreeds. The results suggest that greatest impact on body condition and potentially productivity via milk yield [20] occurs in crossbred animals that are carriers for tick borne apicomplexan parasites. Thus, while these animals may be more likely to survive acute disease, relative to *Bos taurus*, carrier infection may still have significant economic consequences. This suggests that native cattle have greater potential to reduce the pathogenic sequelae of these tick-borne organisms than crossbreeds when singly infected. It is therefore possible that native breeds harbor genetic loci which endow a greater tolerance to infection [34, 35]. In order to fully disentangle and validate these effects, longitudinal studies on a cohort of animals with full assessment of infection status and productivity scores is required.

An important consideration raised by this study concerns the interpretation of results from cross sectional epidemiological surveys. One of the challenges when using data collected from a “real world” situation can be in disentangling the impact of different intrinsic and extrinsic factors when they are not distributed evenly across the sample set. We sampled from a large number of animals across different agro-climatic regions to investigate differences in relative abundance of each TBP, using a statistical approach to investigate any underlying associations. The data clearly showed that there are large differences in the distribution of different TBP in Maharashtra state. However, non-identical farming conditions and non-identical distributions of different host types means that disentangling the causes and consequences of these regional differences are very difficult. A further factor we did not consider in this study was date of sampling: we sampled randomly with respect to time-of-year across zones so it is unlikely that differences in seasonal infections were responsible for generating the differences between zones. Moreover, since we were dealing with carrier status, which can persist over multiple years, across all age classes, so impacts of date within a year were likely to be minimized. To fully understand the factors that cause regional differences in epidemiology and consequences of TBD, future studies should incorporate controlled longitudinal experimental data and carefully matched sample sets or study sites.

Given the estimated burden of tick-borne disease to livestock in India, generation of models to identify the most important factors linked to TBD occurrence and its economic consequences are a priority. These would permit the cost-benefit of control regimes to be investigated and whether to aim for control strategies that generate an infection free herd or a state of endemic infection to protect against clinical disease. The results of this preliminary study indicate that, in particular, there may be potential benefit to preventing the carrier state of tick-borne apicomplexan parasites in crossbred animals; they also suggest that targeted breeding of cattle by selecting for both high productivity and greater resistance/tolerance to tick-borne pathogen infection could provide improved economic performance of cattle for India.

## Supporting information

S1 Table List of universal and species-specific primers used for PCR amplification of 18s rRNA (*Babesia/Theileria*) and 16s rRNA (*Anaplasma*/*Ehrilichia*). (DOCX)

S2 Table Blast matches and sequences for representative PCR products sequenced. (DOCX)

S3 Table TBP co-infections detected in bovine animals in Maharashtra state. (DOCX)

S4 Table Species identity of 100 representative ticks collected at different locations. (DOCX)

S1 Fig Visual guidelines to cattle body condition scoring with manual palpation. (DOCX)

S2 Fig Gel photos from universal or species specific PCRs. (DOCX)

S1 Dataset (XLSX)
