# Supplementary material for: PCR diagnosis of tick-borne pathogens in Maharashtra state, India indicates fitness cost associated with carrier infections is greater for crossbreed than native cattle breeds
Source: PLoS One. 2017 Mar 30;12(3):e0174595. doi: 10.1371/journal.pone.0174595 (PMC5373575; doi:10.1371/journal.pone.0174595)
Supplement: S1 Table — (DOCX) [file pone.0174595.s001.docx]

**S1 Table:** List of universal and species-specific primers used for PCR amplification of 18s rRNA (*Babesia/Theileria*) and 16s rRNA (*Anaplasma*/*Ehrilichia*)

| **Parasite Species** | **Primer Sequence** | **Amplicon size (bp)** | **Reference** |
| --- | --- | --- | --- |
| **PRIMER SET 1** |  |  |  |
| *Theileria/Babesia* universal | F: 5^/^-GACACAGGGAGGTAGTGACAAG-3^/^ R: 5^/^-CTAAGAATTTCACCTCTGACAGT-3^/^ | 385 to 429 | Simuunza et al. (2011) |
| Species specific |  |  |  |
| *T. annulata* | F: 5^/^-ACGGAGTTTCTTTGTCTGA-3^/^ | 193 | Ghaemi et al. (2012) |
| *T. orientalis* | F: 5^/^-ACATTTCTCTTGTTTGAGT-3^/^ | 235 | Ghaemi et al. (2012) |
| *B. bovis* | R: 5^/^-GCTCAATTATACAGGCGAAACCTGC-3^/^ | 278 | Simuunza et al. (2011) |
| *B. bigemina* | R: 5^/^-CGTTCCTATTAACCATTACCAAGGCTCA-3^/^ | 359 | Simuunza et al. (2011) |
| **PRIMER SET 2** |  |  |  |
| *Anaplasma/Ehrlicha,* universal | F: 5^/^-GGTTTAATTCGATGCAACGCGA-3^/^ R: 5^/^-CGTATTCACCGTGGCATG-3^/^ | 430 | Simuunza et al. (2011) |
| Species specific |  |  |  |
| *Anaplasma spp.* | R: 5^/^-GCTCAGCCTTGCGACGTT-3^/^ | 335 | Simuunza et al. (2011) |
| *E. ruminantium* | R: 5^/^-GAGTGCCCAGCATTACCTGT-3^/^ | 201 | Simuunza et al. (2011) |
