# Supplementary material for: PCR diagnosis of tick-borne pathogens in Maharashtra state, India indicates fitness cost associated with carrier infections is greater for crossbreed than native cattle breeds
Source: PLoS One. 2017 Mar 30;12(3):e0174595. doi: 10.1371/journal.pone.0174595 (PMC5373575; doi:10.1371/journal.pone.0174595)
Supplement: S2 Table — (DOCX) [file pone.0174595.s002.docx]

**S2 Table: Blast matches and sequences for representative PCR products sequenced**

| **Intended Product** | **Cleaned Sequence** | **Top Blast Hit/Accession number** | **% Identical** |
| --- | --- | --- | --- |
| *T. annulata* 1 | GAGTGCTCAAGCATGCTTTCGCCTTGATAGTTTAGCATGGAATAATAAAGTAGGACTTTGGTTCTATTTTGTTGGTTTTAGGTACCAAAGTAATGGTTAATAGGAACAGTTGGGGGCATTCGTATTTAACTGTCAGAGGTGAAATTCTTAGA | *T. annulata* [LC094960.1](https://www.ncbi.nlm.nih.gov/nucleotide/953514470?report=genbank&log$=nucltop&blast_rank=1&RID=BDGYDU5H01R) | 99% |
| T. annulata 2 | AAGACAGTGCTTTCGCCTTGAATAGTTTAGCATGGAATAATAAAGTAGGACTTTGGTTCTATTTTGTTGGTTTTAGGTACCAAAGTAATGGTTAATAGGAACAGTTGGGGGCATTCGTATTCAACTGTCAGAGGTGAAATTCTTAGAC | *T. annulata* [KT367867.1](https://www.ncbi.nlm.nih.gov/nucleotide/929984467?report=genbank&log$=nucltop&blast_rank=1&RID=BDHFCFYF016) | 100% |
| *T. orientalis 1* | CCGGAGATTACTTTGAAAATTAAATTGCCCAAAGCAGGCTTTTGCCTTGAATATTTTACCATGGAATAATAAATAGGACTTTGGTTCAATTTTGTTGGTTTTAGGTACCAAA | *Theileria** [FJ595120.1](https://www.ncbi.nlm.nih.gov/nucleotide/220172381?report=genbank&log$=nucltop&blast_rank=1&RID=BDJA27R0013) | 92% |
| *T. orientalis 2* | CCGGAGTATTACTTTGAGAGAATTAAATTGCCCAAAGCAGGCTTTTGCCTTGAATATTTTAGCATGGAATAATAAAGTAGGACTTTGGTTCTATTTTGTTGGTTTTAGGTACCAAAGTAAGGGTTAAAAGGAACAGTTGGGGGCATTCTTATTTAACTGTCAAAGGTGAAATT | *T. orientalis* [KU958549.1](https://www.ncbi.nlm.nih.gov/nucleotide/1042773261?report=genbank&log$=nucltop&blast_rank=2&RID=BDVEAU96013) | 94% |
| *B. bovis 1* | TAAAACTACGAGCTTTTTAACTGCAACAAGTTTAATATACGCTATTGGAGCTGGAATTACCGCGGCTGCTGGCACCAGACTTGCCCTCCAATGGGTACTCGGGCGAGGGTGAAGGTCGCCCCCATGCCAATTACAGAGCAGTAGCCCCGTATTGGTATTTCTGGTCACTACCTCCCTGTGT | *B. bovis* [KP745628.1](https://www.ncbi.nlm.nih.gov/nucleotide/803341935?report=genbank&log$=nucltop&blast_rank=1&RID=BDVGMH0F013) | 99% |
| *B. bovis 2* | As above | As above | As above |
| *B. bigemina* | GCTGAAGTATTCAAGACAAAAGTCTGCTTGAAACACTCTAATTTTCTCAAAGTAAAAAAAAAGCCAGCGAAAAGACCCAAAACCAGGGAAAAAACGCGAGGCTGAAATACAACTACGAGCTTTTTAACTGCAACAAGTTTAATATACGCTATTGGAGCTGGAATTACCGCGGCTGCTGGCACCAGACTTGCCCTCCAATTGGTACTCTGGTGAGGTTGTACATCACCATCATTCCAATTACAAGACGAAAGCCCTGTATTGTTATTTCTTGTCACTACCTCCCTGTG | *B. bigemina* [KU206297.1](https://www.ncbi.nlm.nih.gov/nucleotide/1026817123?report=genbank&log$=nucltop&blast_rank=1&RID=BDVM3TYM013) | 100% |
| *B. bigemina 2* | As above | As above | As above |
| *Anaplasma 1* | CGTTGCTGCTGGAGGCTAGATCCTTCTTAACAGAAGGGCGCAGTTCGGCTGGGCCTCGCACAGGTGCTGCATGGCTGTCGTCAGCTCGTGTCGTGAGATGTTGGGTTAAGTCCCGCAACGAGCGCAACCCTCATCCTTAGTTACCAACAGGTAATGCTGGGCACTACGCCCCCCGTTTCCCCTGGTACCTCAGTTGCTGCACAATAAAACAAATCACGTAGCTGGTCACTCAACACGGGGGCGCTC | *A. marginale* [KU686785.1](https://www.ncbi.nlm.nih.gov/nucleotide/1035287350?report=genbank&log$=nucltop&blast_rank=3&RID=BDXJ5NPF013) | 98% |
| *Anaplasma 2* | CCGTGCTGACTTGACATCATCCCCACCTTCCTCCAGTTTACCACTGGCAGTCTCCTTAAAGTGCCCGGCTTAACCCGCTGGCAACTAAGGATGAGGGTTACGCTCGTTGCGGGACTTAACCCAACATCTCACGACACGAGCTGACGACAGCCATGCAGCACCTGTGCGAGATCCAGCCGAACTGCGCCCTTCTGTTAAGAAGGATCTAATCTCCATGTCAAGAAGTGGTAAGGTTCTTCGCGTTGCATC | *A. cameli*** [KX765882.1](https://www.ncbi.nlm.nih.gov/nucleotide/1092916538?report=genbank&log$=nucltop&blast_rank=1&RID=BDXZP50N016) | [100%](https://www.ncbi.nlm.nih.gov/nucleotide/1092916538?report=genbank&log$=nucltop&blast_rank=1&RID=BDXZP50N016) |
| *E. ruminantium 1* | TCATCGCACGACACGTAGCTGACGTACTGCCATGCTGCACCTGTGTAAACGACCAGCCGAACTGCGCCCTTCTGTTAAGAAGGATCTAGCCTCCATGTCAAGAAGTGGTAAGGTTTTTCGCGTTGCATCGAATTAAACC | *A. marginale* [KU686785.1](https://www.ncbi.nlm.nih.gov/nucleotide/1035287350?report=genbank&log$=nucltop&blast_rank=5&RID=BDX27ZKX013) | 93% |
| *E.ruminantium 2* | CATGTTGCACCTGTGCGACGACCAGCCGAACTGCGCCCTTCTGTTAAGAAGGATCTAGCCTCCATGTCAAGAAGTGGTAAGGTTTTTCGCGTTGCATCGAATTAAACCC | *A. marginale* [KU686785.1](https://www.ncbi.nlm.nih.gov/nucleotide/1035287350?report=genbank&log$=nucltop&blast_rank=6&RID=BDX855VV013) | 96% |

***for this isolate the top BLAST match was an unnamed *Theileria* sp. isolate, but *T orientalis* was listed as next best match with 91% identity: placement in the *T. orientalis/ buffeli* complex is most likely**

****A. camelli not currently an officially recognised species but see** H. Ait Lbacha, Z. Zouagui, S. Alali, A. Rhalem, E. Petit, M. J. Ducrotoy, H.-J. Boulouis and R. Maillard. “Candidatus anaplasma camelii” in one-humped camels (Camelus dromedarius) in Morocco: a novel and emerging anaplasma species? Infectious Diseases of Poverty. 2017, 6:1. DOI: 10.1186/s40249-016-0216-8.
