# Supplementary material for: PCR diagnosis of tick-borne pathogens in Maharashtra state, India indicates fitness cost associated with carrier infections is greater for crossbreed than native cattle breeds
Source: PLoS One. 2017 Mar 30;12(3):e0174595. doi: 10.1371/journal.pone.0174595 (PMC5373575; doi:10.1371/journal.pone.0174595)
Supplement: S3 Table — (DOCX) [file pone.0174595.s003.docx]

**S3 Table TBP Co-infections Detected In Bovine Animals in Maharashtra State**

|  | Total Number of Infected Animals | Number of Single Infections | Number Co-infections *T annulata** | Number Co-infections  *T orientalis** | Number Co-infections *Babesia* spp.* | Number Co-infections *Anaplasma*  spp.* | Number Double Infections | Number Triple Infections | Number >3 Infections |
| --- | --- | --- | --- | --- | --- | --- | --- | --- | --- |
| ***T. annulata*** | 166 | 43 | N/A | 70 | 31 | 90 | 60 | 47 | 16 |
| ***Anaplasma* spp.** | 561 | 445 | 98 | 65 | 33 | N/A | 53 | 47 | 16 |

The table displays information on the breakdown of co-infection status for the two most commonly detected tick-borne pathogens: *Theileria annulata* and *Anaplasma* spp. For each of these pathogen types the number of single, double and triple or more infections is provided. Additionally, a breakdown of the number of times co-infection occurred with one of the other detected pathogen types is provided: please note, cases denoted * are not exclusive double infections i.e. animals marked as Co-infection *T. annulata* with *T orientalis* may also have been infected with additional pathogen species/genera. Therefore, total number of infected animals for the two most common pathogens (column 1) is computed by: adding the number of single, double, triple and other (>3) infections. *Babesia* was uncommon in our survey so for simplicity and clarity *Babesia bovis* and *Babesia bigemina* have been grouped as *Babesia* spp. in the table.
