# Supplementary material for: PCR diagnosis of tick-borne pathogens in Maharashtra state, India indicates fitness cost associated with carrier infections is greater for crossbreed than native cattle breeds
Source: PLoS One. 2017 Mar 30;12(3):e0174595. doi: 10.1371/journal.pone.0174595 (PMC5373575; doi:10.1371/journal.pone.0174595)
Supplement: S4 Table — (DOCX) [file pone.0174595.s004.docx]

**S4 Table: Species Identity of 100 representative ticks collected at different locations**

| **Agroclimatic Zone** | **No of Ticks Collected** | **Tick** | | |
| --- | --- | --- | --- | --- |
|  |  | ***Rhipicephalus (Boophilus) microplus*** | ***Rhipicephalus***  ***haemaphysaloides*** | ***Hyalomma anatolicum anatolicum*** |
| **Deori,** [**Eastern Vidarbha Zone**](http://www.mahaagri.gov.in/CropWeather/AgroClimaticZone.html#evz)  **Dist Gondiya (M.S) India**  **GPS N: 21. 20 009. E: 80. 22657** | **100** | **70** | **10** | **20** |
| **Akot,** [**Assured Rainfall Zone**](http://www.mahaagri.gov.in/CropWeather/AgroClimaticZone.html#arz)  **Dist Akola , (M.S) India**  **GPS N: 21. 05 0771. E: 077. 04494** | **100** | **73** | **-** | **37** |
| **Nagpur,** [**Moderate Rainfall Zone**](http://www.mahaagri.gov.in/CropWeather/AgroClimaticZone.html#mrz)**Dist Nagpur, (M.S) India**  **GPS N: 21. 9′ 0 E: 79. 5′ 24**  **Samples from clinical cases** | **100** | **70** | **-** | **30** |
| **Udgir,**[**Assured Rainfall Zone**](http://www.mahaagri.gov.in/CropWeather/AgroClimaticZone.html#arz)  **Dist Latur, (M.S) India**  **GPS N: 18.396111 E: 77.1175**  **.** | **100** | **64** | **-** | **36** |
| **Nashik, Scarcity**  [**Zone**](http://www.mahaagri.gov.in/CropWeather/AgroClimaticZone.html#arz)  **Dist Nashik, (M.S) India**  **N: 20.01001 E: 73.7972** | **100** | **25** | **6** | **69** |
