# Supplementary material for: PCR diagnosis of tick-borne pathogens in Maharashtra state, India indicates fitness cost associated with carrier infections is greater for crossbreed than native cattle breeds
Source: PLoS One. 2017 Mar 30;12(3):e0174595. doi: 10.1371/journal.pone.0174595 (PMC5373575; doi:10.1371/journal.pone.0174595)
Supplement: S1 Fig — (DOCX) [file pone.0174595.s005.docx]

**S1 Fig: Visual Guidelines to cattle body condition scoring with manual palpation (Following recommended protocol of Queensland Government, Australia** [**https://www.daf.qld.gov.au/__data/assets/pdf_file/0015/53520/Animal-HD-Investigation-Condition-scores.pdf**](https://www.daf.qld.gov.au/__data/assets/pdf_file/0015/53520/Animal-HD-Investigation-Condition-scores.pdf) **)
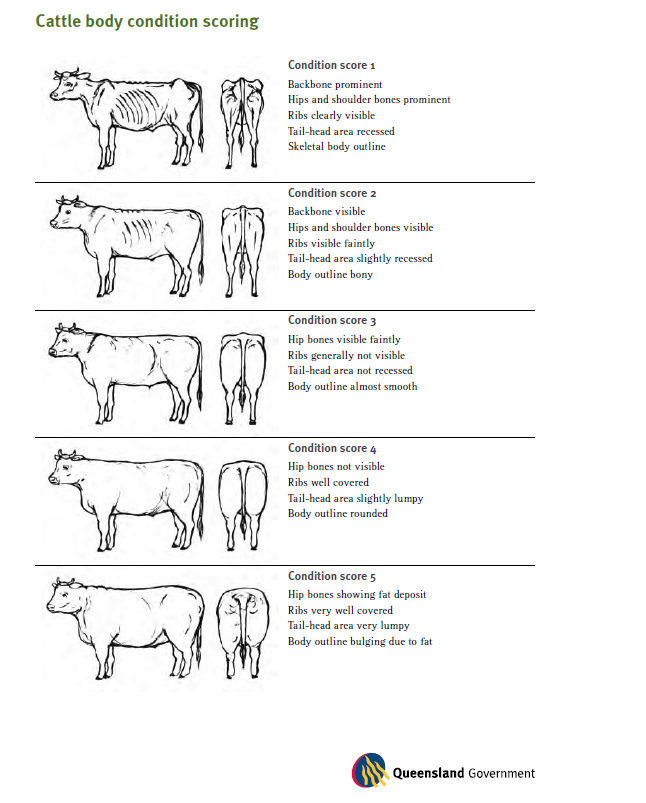
**
