# Supplementary material for: PCR diagnosis of tick-borne pathogens in Maharashtra state, India indicates fitness cost associated with carrier infections is greater for crossbreed than native cattle breeds
Source: PLoS One. 2017 Mar 30;12(3):e0174595. doi: 10.1371/journal.pone.0174595 (PMC5373575; doi:10.1371/journal.pone.0174595)
Supplement: S2 Fig — (DOCX) [file pone.0174595.s006.docx]

**S2 Fig: Gel Photos from Universal or Species Specific PCRs.**

A


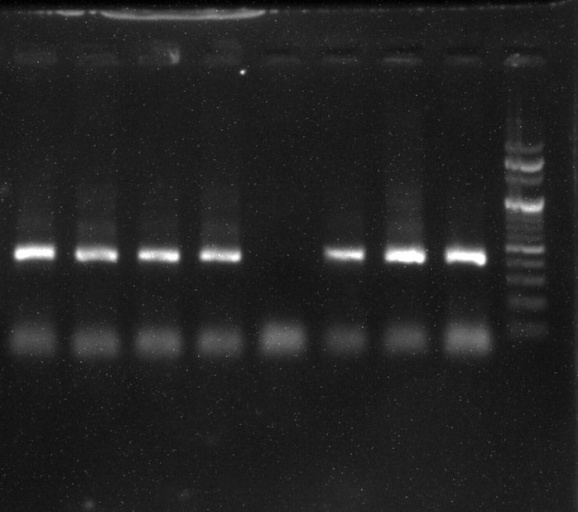


B


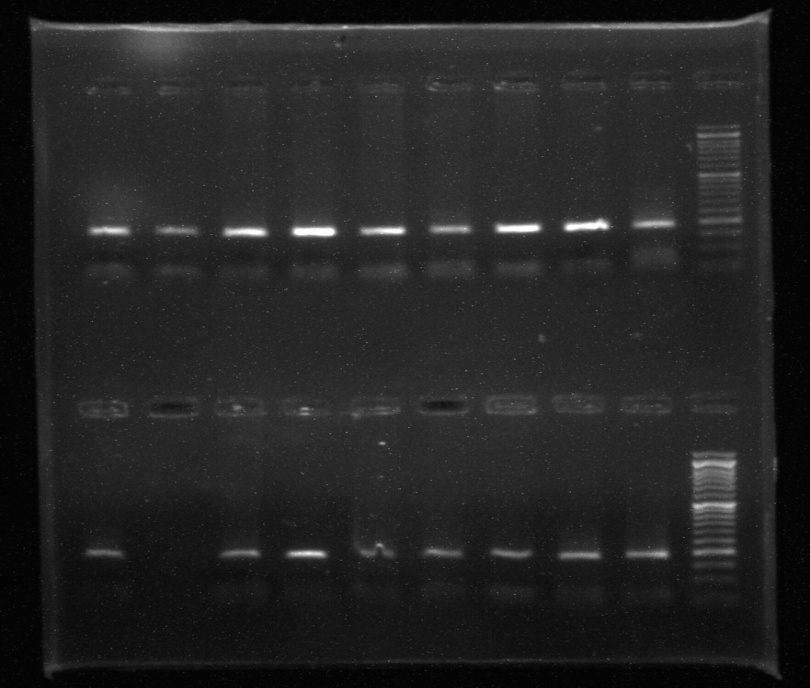


C


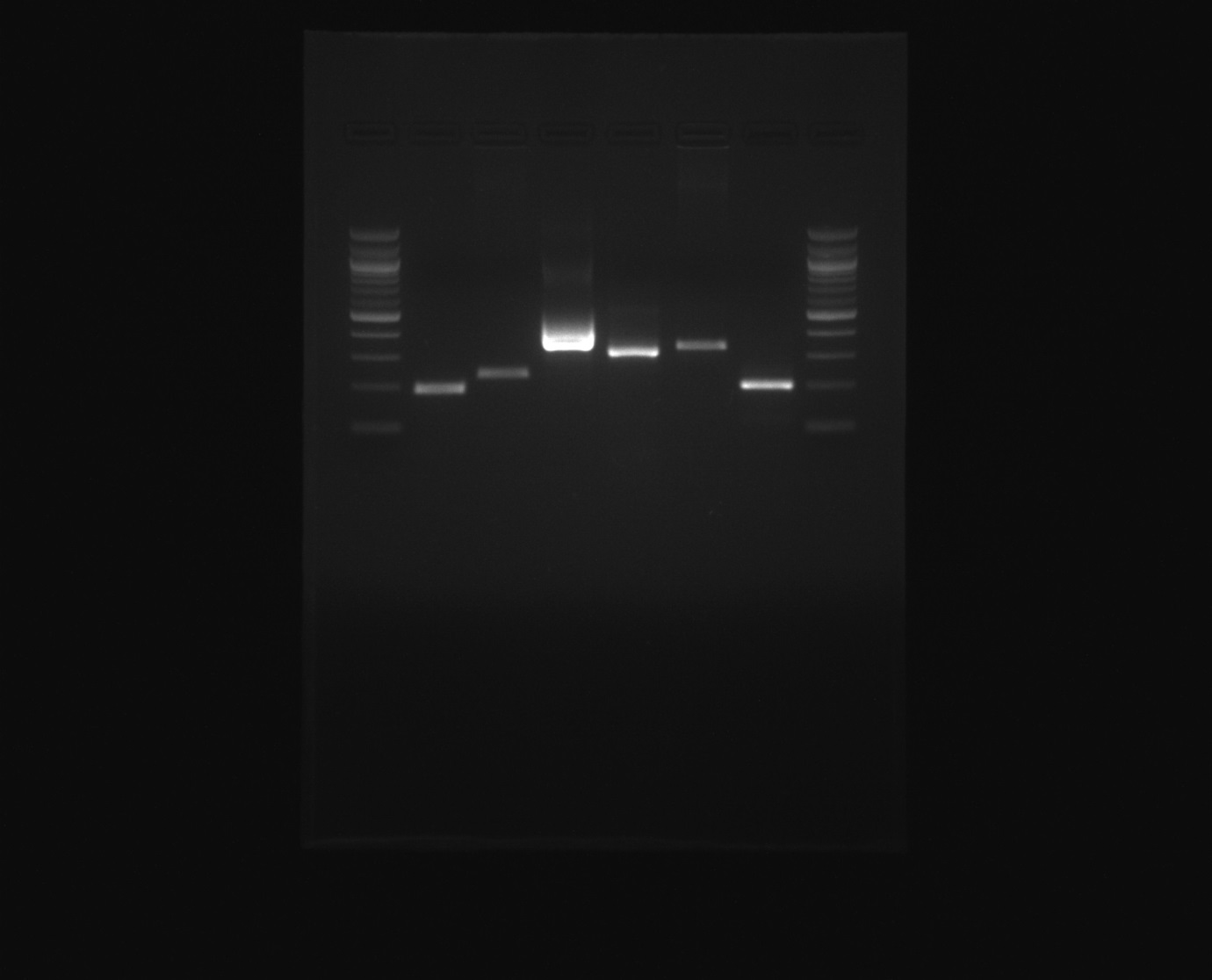


A. Amplicon generated using general primers (Simuunza et al. 2011) for Anaplasma/Ehrlichia. The negative control is 5 from the left. B. Amplicon generated using general primers (Simuunza et al. 2011) for Apicomplexa. The negative control is 2 from the left on the bottom row. C. Amplicons generated for each of the species-specific secondary reactions (Simuunza et al. 2011, Ghaemi et al. 2012): analysis by gel electrophoresis of semi-nested 18s rRNA and 16s rRNA PCR amplification products for Apicomplexa and Anaplasma spp. or Ehrlichia, respectively. Single bands of different sizes, specific for each primer set were obtained. Validation of species specificity was by direct sequencing of the PCR products and BLAST analysis of two generated sequences (See S2 table above) Each lane represents a reference PCR product specific for each parasite tested: lane 1&8, 100bp marker; lane 2, *Theileria annulata*; lane 3, *Theileria orientalis*; lane 4, *Babesia bigemina*; lane 5, *Babesia bovis*; lane 6, *Anaplasma*; lane 7, primer set for *Ehrlichia ruminantium (Anaplasma* sp. validated).
